# Supplementary material for: Barriers and enablers to access childhood cataract services across India. A qualitative study using the Theoretical Domains Framework (TDF) of behaviour change
Source: PLoS One. 2021 Dec 31;16(12):e0261308. doi: 10.1371/journal.pone.0261308 (PMC8719670; doi:10.1371/journal.pone.0261308)
Supplement: S1 File — (DOCX) [file pone.0261308.s001.docx]

**S1 File. Topic guide for in-depth interviews with parents**

*Introduction*

- Thank participants for agreeing to take part
- Do you have any questions regarding our information sheet?
- Explain that this session will be aimed at getting their views about the issues involved in accessing cataract services for children
- Explain that the session is about their views in general along with their personal experiences with their child
- Session will last approximately 1.5 hours

*Respondent introductions*

- Please tell me a bit about yourself / your family/ age range of the respondent and spouse/ education and occupation of both parents/ how many children you have and their ages/

*Knowledge about childhood cataract*

1. What is cataract?
2. How might cataract affect a child’s vision and/or their life?
3. Is cataract in children preventable or treatable? If so how?
4. What are some of the main reasons on why some children develop cataract?
5. Are there any self-practices adopted for treating cataract in your communities?

*Attitude to accessing childhood cataract services*

1. When do you think the child with cataract should be taken to an eye specialist?
2. At what age can a child undergo surgery for cataract? Would it be ok to delay surgery until the child is grown up?
3. What is the decision making process in your household for accessing eye health services?
4. What are the risks and benefits for a child to undergo cataract surgery?
5. Is cataract surgery the end of the treatment? If yes, why do you think so? If not what else is required
6. Is there any gender disparity in accessing the services?

*Probe on:*

- Follow up period / care required
- Issues in attending follow up care

1. Are there any problems in accessing cataract services for children? Explain at the family, community and at the hospital level

*Probe on:*

- Access related
- Affordability
- Dependability / dependants
- Fear
- Cultural reasons etc.
- Problems at the Hospital
- Any special foods good for eye health

1. How to ensure the children with cataract completes their treatment on time?

*Probe on:*

- Whose responsibility is this?
- What is needed?
- The child his / her condition

1. Can you please explain about your child’s eye problem?

Probe on

- Child’s age
- What was the problem?
- When it was noticed? By whom?
- Did anyone else in the family has similar problem?
- What did you do on recognition of the problem?
- Surgery status – when was surgery completed? Prognosis: what will happen in the future – will the condition develop, improve, and deteriorate?
- If surgery not completed – why?
- Finally, is there anything you would like to add about this topic before concluding the interview?

END
