# Supplementary material for: Barriers and enablers to access childhood cataract services across India. A qualitative study using the Theoretical Domains Framework (TDF) of behaviour change
Source: PLoS One. 2021 Dec 31;16(12):e0261308. doi: 10.1371/journal.pone.0261308 (PMC8719670; doi:10.1371/journal.pone.0261308)
Supplement: S1 Table — (DOCX) [file pone.0261308.s002.docx]

| S1 Table. Summary of TDF domains for Barriers and Enablers | | | |  |
| --- | --- | --- | --- | --- |
| **Barriers to access childhood cataract services ^a^** | | | |  |
| **TDF Domains** | **Rank order** | **Frequency of utterances** | **Elaboration ^b^** | **% of total utterances** |
| Environmental context and resources | 1 | 77 | 9 | 24% |
| Beliefs about consequences | 2 | 74 | 9 | 23% |
| Social influences | 3 | 48 | 8 | 15% |
| Emotions | 4 | 35 | 6 | 11% |
| Motivation and goals | 5 | 27 | 4 | 9% |
| Knowledge | 6 | 24 | 2 | 8% |
| Beliefs about capabilities | 7 | 11 | 3 | 3% |
| Nature of behaviour | 8 | 10 | 4 | 3% |
| Behaviour regulation | 9 | 3 | 1 | 1% |
| Skills | 10 | 4 | 1 | 1% |
| Social professional role and identity | 11 | 2 | 1 | 1% |
| Memory, attention and decision processes | N/A | 0 | 0 | 0% |
| Total |  | 317 | 48 |  |
|  |  |  |  |  |
| **Enablers to access childhood cataract services ^a^** | | | |  |
| **TDF Domains** | **Rank order** | **Frequency of utterances** | **Elaboration ^b^** | **% of total utterances** |
| Social influences | 1 | 68 | 7 | 21% |
| Beliefs about consequences | 2 | 67 | 10 | 20% |
| Motivation and goals | 3 | 59 | 6 | 18% |
| Knowledge | 4 | 40 | 6 | 12% |
| Beliefs about capabilities | 5 | 22 | 2 | 7% |
| Emotions | 6 | 21 | 3 | 6% |
| Environmental context and resources | 7 | 17 | 4 | 5% |
| Behaviour regulation | 8 | 16 | 3 | 5% |
| Social professional role and identity | 9 | 9 | 2 | 3% |
| Nature of behaviour | 10 | 6 | 3 | 2% |
| Skills | 11 | 3 | 2 | 1% |
| Memory, attention and decision processes | N/A | 0 | 0 | 0% |
| Total |  | 328 | 48 |  |
| **^a^** The TDF domains accounting for 70% of all utterances in either barrier or enabler categories are presented above the double lines  ^b^ Includes number of themes and sub themes identified | | | |  |
