# Supplementary material for: Barriers and enablers to access childhood cataract services across India. A qualitative study using the Theoretical Domains Framework (TDF) of behaviour change
Source: PLoS One. 2021 Dec 31;16(12):e0261308. doi: 10.1371/journal.pone.0261308 (PMC8719670; doi:10.1371/journal.pone.0261308)
Supplement: S2 Table — (DOCX) [file pone.0261308.s003.docx]

| S2 Table. Summary of statements classified as barriers, sorted by TDF Domain ***(P – Parents; M – Mother; F – Father; GP – Grandparents; FG –Family group)*** | | | | |
| --- | --- | --- | --- | --- |
| **TDF Domain** | **Identified Theme** | **Frequency (*n* transcripts)** | **Interviewees (Roles, *n*)** | **Sample Quotes** |
| Beliefs about consequences | Outcome Expectancies | 3 | P, GP, FG | It is good to go for surgery if he is cured. Surgery should do good … but they said that the white spots won’t go in his eyes. So why to go for the surgery …. That’s why I didn’t go again (ref Id 27) |
|  | Attitudes | 10 | P (4), F (3),  M (2), FG(1) | No…. we never thought the child should go for an eye check-up when there is no problem... Only if there is a problem, children should be taken for check-up. Otherwise not necessary. (Ref ID 10) |
|  | Beliefs | 16 | P (1), F (11), M (1) GF (2), GP (1) | It is about the faith in god… if god can cure, let him cure… no need to worry (Ref Id 13) |
|  | Critical incidents | 5 | P (1), F (3), FG (1) | She had problem since beginning. She had trouble in seeing. She used to see like this bending her head on one side and squeeze her eyes always. Then we showed her to a local eye doctor. He did not say anything. He only gave number for spectacles. But her discomfort continued. After that we showed her to another doctor who gave medicines to eat… We did all this in our area, where we live. Our local doctor...It is within Delhi… (ref Id 6) |
|  | Unrealistic optimism | 5 | F (2), M (1) FG (1), GP (1) | It happened since he was born. Now he is 4 years old. It is there since four years. We kept on thinking that it will go away, it will be cured. We kept on going to “babaji’s place”. People recommend this place to visit… to get cured. That’s it … and the time kept passing on …(Ref Id 13) |
|  | Regret | 2 | P (1), GP (1) | Yes… patching exercise needs to be done 3-4 times a day….. We also feel that it was probably a wrong decision to do the surgery early. Had it been later, possibly the lens could have been fitted. (ref Id 29) |
|  | Consequences | 19 | F (5), M (6) P (4), GP (4) | We are poor people. We somehow fill our stomach through labour work. How would we know? Local Doctor Sahab told that it will take time. So we came back thinking that let him grow up. Then we will do. (ref Id 26) |
|  | Not a priority | 7 | F (1), M (3) WF (1) | She had it even before but our family did not notice it …She was in class VIII. She used to live in the hostel. She informed us that she can’t see properly... But we laughed it off … she was so young. How could she see less? Nobody understood. Not even me. When it increased, she told us again after 2 months... Then we showed her to the doctor there. (ref Id 16) |
|  | Perceived risk/ threat | 6 | F (2), M (3) P (1) | “The lens will break. That’s why we didn’t do it out of fear. We thought that once he grows up we will do it. We are afraid that the lens might break. (ref ID 26) |
|  | Review | 1 | M (1) | In the government hospital only the doctor’s consultation is free. You have to pay for the medicine. I could arrange some money in three years. Then I went to the hospital. We visited four times. Then they did the surgery. The surgery was done for the nerves. We visited one month after the surgery for follow up. Even after surgery she can't see properly in the operated eye. They called for the next surgery. But we didn’t go… (ref ID 30) |
| Environmental context and resources | Economic | 33 | F (13), M (12) P (7), GP (1) | It took months to recognise the problem in my child. We took the child to the general hospital they asked huge money for the surgery as we don’t have good financial condition, we couldn’t do. We had only yellow ration card which is eligible for only some concession. (ref ID 2) |
|  | Health care facility | 21 | F (10), M (8) P (1), GP (2) | There is no eye hospital near by us, but the local hospital referred us immediately to this eye hospital. The doctors are not well trained at the local hospital also there is no machinery to operate on the child so they sent us here. It will be better if any eye hospital available in our area. (ref ID 2) |
|  | Environmental stressors - Mobility/ transport | 5 | F (1), M (2) P (2) | We live in a village. So we have to come and stay in an acquaintance’s place in the city. We have to start around 4 am in the morning to catch the bus. Then we have to wait for the train… When the train comes … sometimes we miss the train, so we have to wait longer …This time it was very difficult. We were waiting for the bus in the darkness, fog on the roadside … with the child …. The child also caught cold... (ref ID 10) |
|  | Environmental stressors – Distance | 3 | F (2), M (1) P (2) | Yes, it is too far… It takes four hours from Mahbuba to here… Another three hours from Khajuraho. Yes, and it is another 12 kms by vehicle from Khajuraho. (ref ID 13) |
|  | Environmental stressors – Time | 3 | F (2), WF (1) | If there is no problem, who would go for check-up. Who has so much time nowadays…..Only when there is a problem people are ready to go anywhere. (ref ID 28) |
|  | Nature of Environment | 2 | F (1), P (1) | Most important is cleanliness. Many are living in a dirty place and it can cause infections in the eyes. (ref ID 22) |
|  | Person X environment interaction | 8 | F (5), M (1) P (2) | After going from here he told that he wants to go to school. I told to wait for a few days … you never know …. While playing with other children he might get hit. He went one day. He was wearing spectacles. Other children took his spectacles and it broke. Then I stopped sending him again. (ref ID 15) |
|  | Alternative medicine and Traditional practices | 3 | F (1), WF (2) | They said surgery has to be done. I thought it would be cured by homeopathy and treated for 3-4 months... (ref ID 28) |
| Social influences | Social support (lack of concern/ misguidance/ inappropriate advice etc.) | 7 | F (3), M (3) P (1) | I noticed his movements and asked my parents about it – why he walks like that … he would look as if he has crossed eyes. My parents said that your child has crossed eyes that’s why he walks like that. They said that it is a good sign of luck (ref ID 4) |
|  | Social identity | 3 | P (3) | There are people who think like that … even nowadays in my area. Boys receives better care than the girls. (ref ID 18) |
|  | Social pressure | 8 | F (2), M (3) P (2), WF (1) | My daughter would look always downwards, never upwards. I would say there must be some reason that she never looks upwards. But elders at home and many people said that this happens to the children and it will be cured later. (ref ID 10) |
|  | Power / hierarchy | 7 | F (3), M (2) P (1), WF (1) | When he would drink milk, I noticed white spots in the eyes. I told to my mother in law. She said it happens in children. I said no but she was the head (in the family)… and she didn’t agree. (ref ID 14) |
|  | Social norms / culture | 13 | F (5), GP (1) P (6), WF (1) | We felt very sad. She is so young and she has got cataract! What would happen if we get her married? Problems can arise in her marriage life. (ref ID 16) |
|  | Parental negligence | 6 | F (3), GP (1) M (2) | As a grandparent, I take the decision because the parents are upset because the child has got this problem and they are not willing to spend money for the treatment… (ref ID 1) |
|  | Organisation norms | 2 | F (1), M (1) | Till date we never had any eye camp in our villages. We have to depend on these village doctors. We don’t have other options. (ref ID 4) |
|  | Conflicts | 2 | F (1), M (1) | Yes, we have given all immunisation to him. The nurse didn’t say anything about his eye problems. He is eight years now and he does not go to school. He used to go. But due to this problem he could not see and stopped going. (ref ID 31) |
| Motivation and goals | Certainty of intention | 11 | F (4), P (4) M (3) | First thing… I did not want my child to be operated upon as he is too small. If something happens … I had never before known or seen such a small child being operated. That’s why I never thought of getting the surgery done. (ref ID 4) |
|  | Priority | 7 | F (3), P (2) M (1), GP (1) | They told to come after 2 months... we have to see ... there is no time, expenses too and we have to close the shop... it gets more difficult for other two daughters to go to school (ref ID 12) |
|  | Intention | 6 | F (3), M (1), WF (2) | People also think that if we go to hospital, the whole day will go and the day’s earning will be lost. So our routine will be disturbed…. This is also a reason. (ref ID 6) |
|  | Goals and motivation | 3 | P (1), M (1), GP (1) | Two times camp was organized. I got two of my children treated through the camp. I didn’t go to hospital directly as I had no money.(ref ID 20) |
| Emotions | Effect | 8 | F (3), M (3), GP (2) | I don’t feel good that a lens could not be fitted. Doctor has suggested exercise. But he [the child] doesn’t let us to get him to do those…as he is unable to see with one eye at all. The doctor didn’t tell us about it earlier the lens couldn’t be fitted … we don’t know what the problem in putting the lens… now he needs to use contact lens permanently. Until now he must have lost 10-15 lenses (ref ID 29) |
|  | Stress | 8 | F (5), M (2), P (1) | His father is doing labour job … Here we have difficulty in getting food and now all this … you should understand. There is no rain, only dry taps. No water… (ref ID 26) |
|  | Anxiety/ Depression | 8 | F (1), M (3), P (3), GP (1) | I felt very bad. I cried a lot for two-three days. Such a small child, what has happened to her eyes, how we would manage … we thought all these and cried. (ref ID 10) |
|  | Fear | 5 | F (2), M (2), P (1) | I felt very scared about surgery… Because she is very young …(ref ID 12) |
|  | Anticipated regret | 4 | F (1), M (1), P (1), WF (1) | I didn’t realise so far…If he cannot see then I must get him treated. Otherwise when he grows up he will blame us that we spoiled his eye. (ref ID 27) |
|  | Burnout | 2 | M (1), P (1) | Coming by train becomes a bit difficult at times…. From that far. We have to come in the general compartment. Sometimes it is crowded, sometimes it is comfortable… Money … well … we are poor anyway … we have to arrange for money by doing labour work …There was no money so he went out for two months and earned money. (ref ID 14) |
| Skills | Competence of local doctor/ nurse/ school eye health team | 4 | F (3), GP (1) | We also asked the doctor with whom we regularly consult for him whether he has any problem in the eyes. He said no there is nothing to worry. No. They say that new born babies have blurred vision up to 20 days …..(ref ID 29) |
| Social professional role and identity | Organisation role | 2 | M (1), F (1) | Camps are organized in our area. But these are done once in two months or six months or one year (no regularity). The second thing is, in these camps they don’t talk about children’s problems. Mostly the elderly people que up and they do not pay attention to the children. (ref ID 6) |
| Behaviour regulation | Barriers to Goal | 3 | GP (1), F (2) | Now that surgery is suggested, then if spectacles are required after surgery…. The child can’t wear spectacles or will not be able to protect it. So it will be better to do the surgery after 5-6 years. (ref ID 5) |
| Beliefs about capabilities | Perceived competence | 8 | F (4), M (1), P (1), GP (2) | She used to squeeze her eyes…… roughly at the age of three years. We thought it’s not normal so We searched for a hospital….Just for the normal eye check-up …. We went to the nearest eye specialist and We went in the same week. They could not detect the cataract. They just gave spectacle prescription. We doubted and went to other hospitals ….(ref ID 22) |
|  | Perceived competence on government hospital | 2 | F (1), M (1) | We go to the government hospital, they say go to that place with this slip, or go to that doctor. And in our place if someone goes to the hospital, for one report nobody knows how many hours or days it will take. (ref ID 5) |
|  | Optimism | 1 | P (1) | People said about Ayurveda [other form of medicine based on plants and herbs] treatment. We had tried with Ayurveda in parallel and came to know that by applying Ayurveda medicines in the eyes cataract could be cleared... (ref ID 18) |
| Nature of behaviour | Direct experience | 6 | F (3), M (2), WF (1) | We brought him once when he was even younger. He had cough. We were told that surgery cannot be done. So I thought, surgery cannot be done in very small children. (ref Id 13) |
|  | Past behaviour | 1 | F (1) | Yes, there are these small capsules... We used to put those if there is redness or dust falls in the eyes. (ref Id 16) |
|  | Routine habit | 2 | WF (28), F (1) | For weakness etc. we take medicine from the medical shops. If there is fever, we go to the nearby hospital. (ref Id 31) |
|  | Breaking habit | 1 | F (1) | We always try to give healthy food to the children. But nowadays children also give trouble. They won’t eat. If their mother cooks something, they might not eat. Some would ask for Maggi, some would ask for bread. (ref Id 6) |
| Knowledge | Knowledge about condition | 19 | F (9), M (5), P (4), WF (1) | Yes, I have heard about cataract before. Often older people get cataract surgery. So I knew about cataract. But, I never heard or saw cataract in children. (ref Id 10) |
|  | Knowledge | 5 | F (4), P (1) | In the villages, many people don’t know that children having eye problems should be taken to the hospital. There is no information. (ref Id 16) |
