# Supplementary material for: Barriers and enablers to access childhood cataract services across India. A qualitative study using the Theoretical Domains Framework (TDF) of behaviour change
Source: PLoS One. 2021 Dec 31;16(12):e0261308. doi: 10.1371/journal.pone.0261308 (PMC8719670; doi:10.1371/journal.pone.0261308)
Supplement: S3 Table — (DOCX) [file pone.0261308.s004.docx]

| S3 Table. Summary of statements classified as enablers, sorted by TDF Domain ***(P – Parents; M – Mother; F – Father; GP – Grandparents; FG –Family group)*** | | | | |
| --- | --- | --- | --- | --- |
| **TDF Domain** | **Identified Theme** | **Frequency (*n* transcripts)** | **Interviewees (Roles, *n*)** | **Sample Quotes** |
| Beliefs about consequences | Outcome Expectancies | 6 | GP (2), P (3), M (1) | It’s not a good idea to delay the surgery. I feel the eye will get weaker if the surgery is delayed. It is better to get operated at the right time for a better vision. (ref Id 22) |
|  | Attitudes | 17 | GP (1), P (8), M (1), F (7) | We felt … but, then we thought it is about the eyes and eyes are everything. So it is necessary. My elder brother also came. We quickly took the decision to go ahead with the surgery and admitted her. (ref Id 10) |
|  | Reinforcement | 11 | GP (1), P (3), M (1), F (6) | The doctors told that the surgery should be done immediately as with time the situation of the child’s eye sight would worsen. They showed me children aged as little as 4 months on whom surgery had been done and tried to convince me. They said that it is quite normal. After knowing all this, I became confident and went ahead with the surgery. (ref Id 4) |
|  | Beliefs | 3 | GP (1), F (2) | Cataract is not god’s curse. In many people after cataract surgery they can see. I believe in god and pray every day in the morning and I believe god blesses us…(ref Id 1) |
|  | Perceived risk/threat | 8 | GP (1), P (3), F (4) | We wanted to get it done at the earliest possible. I also heard that cataract bursts. So we wanted to do it at the earliest. (ref Id 10) |
|  | Priority | 9 | M (3), P (2), F (4) | Yes, I think there is still some treatment to be continued after surgery. We have to come here a few times for her check-up. Well, even if it is a problem coming for follow up, I can't ignore as it’s my responsibility. (ref Id 10) |
|  | Anticipated regret | 1 | F (1) | If he cannot see then I must get him treated. Otherwise when he grows up he will blame us that we spoiled his eye…(ref Id 27) |
|  | Sensitisation | 8 | M (2), P (3), F (2), GP (1) | We can also conduct awareness programs during the fairs at the village. Health exhibitions can be conducted and knowledge on childhood cataract could be given to the villagers. (ref Id 10) |
|  | Appraisal / review | 3 | M (1), P (2) | Before the surgery if we would keep anything, say a toy… in front of them, they would not react. But after the surgery when the bandage was removed, we put a pen in front of him and he immediately grabbed the pen! That was probably the biggest moment of my life … That something is visible now. At that point I felt that we were going in the right direction... (ref Id 18) |
| Environmental context and resources | Health care facility | 4 | P (1), F (2), GP (1) | So when I came here, I had a referral slip with me from the community screening, where it was written ‘contact counter no 4’. So I went to the counter no 4 where the registration was done and then whatever was required these people went ahead with their work and prepared the file. (ref Id 5) |
|  | Person X environment interaction | 9 | P (3), F (4), M (2) | Yes, some people don’t know that even small children could get cataract. Now in our village everyone has come to know. This should be publicized. Now that we know this, we have to tell in our village… also if it comes on TV or newspaper it would be useful. (ref Id 14) |
|  | Environment | 3 | F (1), GP (2) | Publicity can be done through the TV. In villages also everyone watches TV. It is not possible to go to every house and do this. Now if someone comes to us… we can suggest to go to the doctor. Other way is to go to the villages and tell people…(ref Id 29) |
|  | Economic | 1 | F (1) | Yes, I will come as many times the doctor calls for follow-up. Yes, my daughter and wife both come with me .I don’t care about the expenses. INR 100 to come, another 100 to go. It takes about two and half hours by bus and three hours by auto. (ref Id 7) |
| Social influences | Social support | 39 | P (14), F (14), GP (6), M (5) | In the rural areas people don’t have money. So, major decisions are taken after the family members sit together and decide on where the money will be arranged from and how. Sometimes neighbours and / or relatives are also involved. (ref Id 3) |
|  | Social norms / culture | 19 | P (8), F (3), GP (2), M (6) | Whoever is senior in the family takes the decision (ref Id 11) |
|  | Change agents | 10 | P (5), F (2), GP (2), M (1) | Decision nowadays both take together. People have become more aware nowadays. There are so many programs running in the villages, the ASHA worker is there, she keeps on meeting and talking to people. (ref Id 10) |
| Emotions | Fear | 6 | P (1), F (2), GP (2), M (1) | I felt scared…I thought his future should not be wasted. I asked the doctor again. He said that a lens will be fitted and it would cause no harm. Then, I said ok. Let the surgery be done… (ref Id 18) |
|  | Positive effect | 13 | P (5), F (5), M (3) | I did not know about this. They told in the school, I was not there. No I was not scared. I thought that the doctors are there. So why should I fear. So many are there in the hospital. So I did not fear (ref Id 8) |
|  | Anticipated regret | 2 | P (1), F (1) | Not only once. I would suggest yearly once children eyes and all other check-ups should be done. If we ignore and think that everything is fine, it might happen that when there is really a problem we would regret that we should have done it before and control it. (ref Id 18)) |
| Skills | Parents skill in recognition | 2 | P (1), GP (1) | Her mother first noticed something white in the eyes. But I was avoiding thinking that it would go away on its own …(ref Id 10) |
|  | Competence of the hospital staff | 1 | P (1) | If we come to know about anyone with eye problem, we will suggest them to come to this hospital. We have seen that the employees here are very helpful by nature. I have never faced any problem here. Once you reach here, get yourself registered at the counter, then you don’t have to worry for anything further. This is my experience here. (ref Id 10) |
| Social professional role and identity | Social role | 6 | P (5), F (1) | We keep roaming around the villages. So we need to talk about this. So that if anyone is suffering with eye problems, she/he should be taken to the hospital at the earliest. Home remedies should not be used. (ref Id 10) |
|  | Organisation role | 3 | F (1), P (1), GP (1) | There should be camps for the children so that the parents would become aware and the children would get care. It will be good for all. Such camps are never organized. People don’t take children for examination on their own. Because they have no idea that children could have eye problems. They can’t think of it. (ref Id 18) |
| Motivation and goals | Certainty of the intention | 29 | F (10), P (11), GP (3), M(5) | No, never we will arrange marriage for our children within the relatives. Now that we know … We did marry because we didn’t know. (ref Id 18) |
|  | Intention | 18 | F (6), P (6), FG (2), M(4) | We have been told to come every fifteen days. We did not say no. There should not be negligence from our side. His life has to be successful …(ref Id 14) |
|  | Intrinsic motivation / service intention | 8 | F (3), P (3), M(2) | Yes we will go and inform the villagers. Actually this is how it happens in the villages. Whoever knows, has to give proper suggestions or accompany to come here. (ref Id 20) |
|  | Stability of intention | 2 | P (1), M(1) | I don’t know about other things. But it is very important eyes to have regular check-ups, drops etc. We come here once in two months. But in between also if we notice even slight difference or abnormality we get in touch with Sir over phone/WhatsApp and follow his suggestions. If required, we even pay a visit. (ref Id 18) |
|  | Commitment/ Motivation | 2 | P (1), M(1) | 90% of the responsibility lies with us as parents, we have to take the responsibility and take the child to the doctor in time and take the treatment and follow the instructions of the doctor. (ref Id 2) |
|  | Routine | 1 | M (1) | Parents should notice if the children have any difficulty in reading. If the child is keeping the book too close while reading. If the child is having any problem in reading they should immediately take him to the hospital. (ref Id 23) |
| Nature of behaviour | Direct experience | 4 | P (2), F (1), GP (1) | I know in this hospital we can get free treatment so, I came here. (ref Id 1) |
|  | Breaking habit | 1 | F (1) | At home they were saying to put something or other …it might work … desi [indigenous] medicines …No, they told me to go and get it. But there was no guarantee. So I said what the use is if it doesn’t work. So I didn’t use any. (ref Id 15) |
|  | Automatic behaviour | 1 | GP (1) | In our country during the time of birth itself it should be done once by the doctor in the hospital. (ref Id 29) |
| Behaviour regulation | Action planning | 2 | P (2) | Meetings can be arranged in the village to discuss these issues. The meetings could be arranged through the village head or can be conducted in the village primary school where all the parents should be called. These things should be discussed in that meeting. If people are told in such meetings, many people will be aware. (ref Id 10) |
|  | Generating alternatives | 12 | P (4), F (6), GP (1), M (1) | Yes, some people don’t know that even small children could get cataract. Now in our village everyone has come to know. This should be publicized. Now that we know this, we have to tell in our village. Those who are going to other villages should tell the same … That the small children could have problem. Get them checked. People will come to know …. Also if it comes on TV or newspaper it would be useful. (ref Id 14) |
|  | Facilitators | 2 | F (2) | In general hospitals it happens like – go to counter number 3 or go to counter number 4 … and the person goes around in circles. But here you don’t have to do anything. Even the file is also carried by a person. He will make you sit down. After you are seen, the file goes to another place… (ref Id 6) |
| Knowledge | Knowledge about condition | 7 | P (3), F (1), GP (2), M (1) | There was this white coloured thing in his eyes… like a film. So, I could understand that it could be cataract. Got him examined and was confirmed that it was cataract. (ref Id 14) |
|  | Procedural knowledge | 5 | P (3), M (2) | Now we have to implant the lens and this is the first step of the treatment and we need to go forward. Doctor will tell us what needs to be done in the future. (ref Id 2) |
|  | Related Knowledge | 7 | P (3), F (1), GP (2), M (1) | I have heard that we should eat green vegetables and carrots…these things keep the vision good in eyes. (ref Id 6) |
|  | Knowledge on sign and symptoms | 13 | P (1), F (6), GP (2), M (3), FG (1) | She would look only downwards, never upwards. I would say there must be some reason that she never looks upwards... (ref Id 10) |
|  | Awareness about the facility | 2 | P (1), F (1) | For three four years I know about the hospital … The villagers know about it. They told to come here. So I was coming by myself… not through the camp. (ref Id 15) |
|  | New knowledge | 6 | P (2), F (3), GP (1) | I don’t have knowledge about this. But I have seen here that very small children are operated upon. I have seen children as small as one-year-old.(ref Id 6) |
| Beliefs about capabilities | Optimism | 2 | P (1), M (1) | I bring her here for check-up and tests. All four of my children come here for check-ups… (ref Id 20) |
|  | Perceived competence of the doctor | 20 | P (5), F (11), M (4) | Whatever the doctor advise we have to do, doctors have the Jawabdhari [responsibility] so we need to follow their advice. (ref Id 2) |
