# Supplementary figures and images for: Barriers and enablers to access childhood cataract services across India. A qualitative study using the Theoretical Domains Framework (TDF) of behaviour change
Source: PLoS One. 2021 Dec 31;16(12):e0261308. doi: 10.1371/journal.pone.0261308 (PMC8719670; doi:10.1371/journal.pone.0261308)

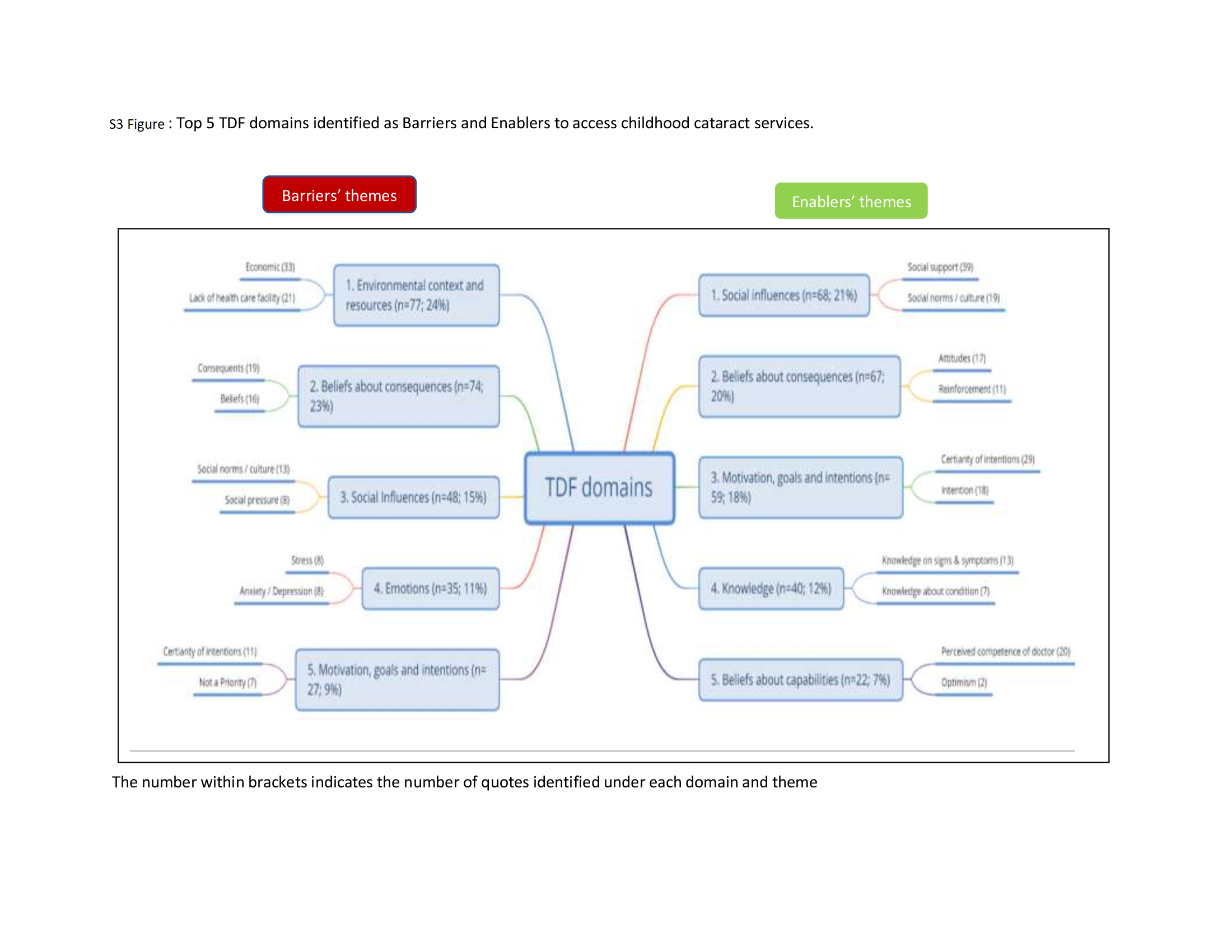

Supplement: S1 Fig — (TIF) [file pone.0261308.s005.tif]
