# Supplementary material for: Barriers and enablers to access childhood cataract services across India. A qualitative study using the Theoretical Domains Framework (TDF) of behaviour change
Source: PLoS One. 2021 Dec 31;16(12):e0261308. doi: 10.1371/journal.pone.0261308 (PMC8719670; doi:10.1371/journal.pone.0261308)
Supplement: Attachment — Submitted filename: PONE-D-21-05307_reviewer (3).pdf [file pone.0261308.s006.pdf]

PLOS ONE

Barriers and enablers to access childhood cataract services across India. A qualitative study using the Theoretical Domains Framework (TDF) of behaviour change

--Manuscript Draft--

Manuscript Number:	PONE-D-21-05307
Article Type:	Research Article
Full Title:	Barriers and enablers to access childhood cataract services across India. A qualitative study using the Theoretical Domains Framework (TDF) of behaviour change
Short Title:	Barriers and enablers to access childhood cataract services across India
Corresponding Author:	Sheeladevi Sethu<br>ORBIS International<br>Gurugram, Haryana INDIA
Keywords:	Childhood cataract, Barriers, Enablers, Theoretical domain framework, Service accessibility
Abstract:	Early presentation for childhood cataract surgery is an important first step in preventing related visual impairment and blindness. In the absence of neonatal eye screening programmes in developing countries, the early identification of childhood cataract remains a major challenge. The primary aim of this study was to identify potential barriers to accessing childhood cataract services from the perspective of parents and carers, as a critical step towards increasing the timely uptake of cataract surgery.<br>Methods:<br>In-depth interviews were conducted using a pre-designed topic guide developed for this study to seek the views of parents and carers in eight Indian states regarding their perceived barriers and enablers to accessing childhood cataract services. A total of 35 in-depth interviews were conducted including 30 at the hospital premises and 5 in the participants' homes.<br>All interviews were conducted in local language and audio taped for further transcription and analysis. Data was organised with NVivo 11 and a thematic analysis was conducted utilising the Theoretical Domains Framework (TDF), an integrative framework of behavioural theories.<br>Results:<br>A total of 632 quotes were coded and a total of 68 themes were identified, including 23 related to barriers, 17 related to enablers and 28 themes represented both barriers and enablers covering 11 out of 12 TDF domains. TDF domains associated with barriers included: 'Environmental context and resources', 'Beliefs about consequences' and 'Social influences'. Reported enablers were identified in three theoretical domains: 'Social influences', 'Beliefs about consequences' and 'Motivations and goals'. This comprehensive TDF approach enabled us to understand the parents perceived barriers and enablers to access childhood cataract services.<br>Conclusion:<br>This study has identified common barriers and enablers to accessing childhood cataract services that could be targeted in future interventions to improve timely uptake.'
Order of Authors:	Sheeladevi Sethu
	John G Lawrenson
	Ramesh Kekunnaya
	Rahul K Ali
	Rishi Raj Borah
	Catherine Suttle
Additional Information:	
Question	Response
Financial Disclosure	NO

Powered by Editorial Manager® and ProduXion Manager® from Aries Systems Corporation

Enter a financial disclosure statement that describes the sources of funding for the work included in this submission. Review the submission guidelines for detailed requirements. View published research articles from *PLOS ONE* for specific examples.<br><br>This statement is required for submission and **will appear in the published article** if the submission is accepted. Please make sure it is accurate.<br><br>**Unfunded studies**<br>Enter: *The author(s) received no specific funding for this work.*<br><br>**Funded studies**<br>Enter a statement with the following details:<br>• Initials of the authors who received each award<br>• Grant numbers awarded to each author<br>• The full name of each funder<br>• URL of each funder website<br>• Did the sponsors or funders play any role in the study design, data collection and analysis, decision to publish, or preparation of the manuscript?<br>• **NO** - Include this sentence at the end of your statement: *The funders had no role in study design, data collection and analysis, decision to publish, or preparation of the manuscript.*<br>• **YES** - Specify the role(s) played.<br><br>* typeset	
**Competing Interests**<br><br>Use the instructions below to enter a competing interest statement for this submission. On behalf of all authors, disclose any competing interests that could be perceived to bias this work—acknowledging all financial support and any other relevant financial or non-financial competing interests.<br><br>This statement is required for submission and **will appear in the published article** if	No authors have competing interests

the submission is accepted. Please make sure it is accurate and that any funding sources listed in your Funding Information later in the submission form are also declared in your Financial Disclosure statement.<br><br>View published research articles from *PLOS ONE* for specific examples.<br><br>**NO authors have competing interests**<br><br>Enter: *The authors have declared that no competing interests exist.*<br><br>**Authors with competing interests**<br><br>Enter competing interest details beginning with this statement:<br><br>*I have read the journal's policy and the authors of this manuscript have the following competing interests: [insert competing interests here]*<br><br>* typeset	
**Ethics Statement**<br><br>Enter an ethics statement for this submission. This statement is required if the study involved:<br><br>• Human participants<br>• Human specimens or tissue<br>• Vertebrate animals or cephalopods<br>• Vertebrate embryos or tissues<br>• Field research<br><br>Write "N/A" if the submission does not require an ethics statement.<br><br>General guidance is provided below. Consult the submission guidelines for detailed instructions. **Make sure that all information entered here is included in the Methods section of the manuscript.**	Ethics clearance obtained from School of Health Sciences at City, University of London and from the local Institutional review board of all 9 participating centers. Written consent was obtained from all study participants

**Format for specific study types**<br><br>**Human Subject Research (involving human participants and/or tissue)**<br>• Give the name of the institutional review board or ethics committee that approved the study<br>• Include the approval number and/or a statement indicating approval of this research<br>• Indicate the form of consent obtained (written/oral) or the reason that consent was not obtained (e.g. the data were analyzed anonymously)<br><br>**Animal Research (involving vertebrate animals, embryos or tissues)**<br>• Provide the name of the Institutional Animal Care and Use Committee (IACUC) or other relevant ethics board that reviewed the study protocol, and indicate whether they approved this research or granted a formal waiver of ethical approval<br>• Include an approval number if one was obtained<br>• If the study involved *non-human primates*, add *additional details* about animal welfare and steps taken to ameliorate suffering<br>• If anesthesia, euthanasia, or any kind of animal sacrifice is part of the study, include briefly which substances and/or methods were applied<br><br>**Field Research**<br><br>Include the following details if this study involves the collection of plant, animal, or other materials from a natural setting:<br>• Field permit number<br>• Name of the institution or relevant body that granted permission	
**Data Availability**<br><br>Authors are required to make all data underlying the findings described fully available, without restriction, and from the time of publication. PLOS allows rare exceptions to address legal and ethical concerns. See the PLOS Data Policy and FAQ for detailed information.	Yes - all data are fully available without restriction

A Data Availability Statement describing where the data can be found is required at submission. Your answers to this question constitute the Data Availability Statement and **will be published in the article**, if accepted.<br><br>**Important:** Stating 'data available on request from the author' is not sufficient. If your data are only available upon request, select 'No' for the first question and explain your exceptional situation in the text box.<br><br>Do the authors confirm that all data underlying the findings described in their manuscript are fully available without restriction?	
**Describe where the data may be found in full sentences. If you are copying our sample text, replace any instances of XXX with the appropriate details.**<br><br>• If the data are **held or will be held in a public repository**, include URLs, accession numbers or DOIs. If this information will only be available after acceptance, indicate this by ticking the box below. For example: *All XXX files are available from the XXX database (accession number(s) XXX, XXX.).*<br>• If the data are all contained **within the manuscript and/or Supporting Information files**, enter the following: *All relevant data are within the manuscript and its Supporting Information files.*<br>• If neither of these applies but you are able to provide **details of access elsewhere**, with or without limitations, please do so. For example:<br><br>*Data cannot be shared publicly because of [XXX]. Data are available from the XXX Institutional Data Access / Ethics Committee (contact via XXX) for researchers who meet the criteria for access to confidential data.*<br><br>*The data underlying the results presented in the study are available from (include the name of the third party*	All relevant data are within the manuscript and its supporting information files

Powered by Editorial Manager® and ProduXion Manager® from Aries Systems Corporation

and contact information or URL).<br>• This text is appropriate if the data are owned by a third party and authors do not have permission to share the data.<br><br>* typeset	
Additional data availability information:	Tick here if your circumstances are not covered by the questions above and you need the journal’s help to make your data available.

Powered by Editorial Manager® and ProduXion Manager® from Aries Systems Corporation

Manuscript

Click here to access/download;Manuscript;Final_Barriers and enablers manuscript.docx

Barriers and enablers to access childhood cataract services across India. A qualitative study using the Theoretical Domains Framework (TDF) of behaviour change

Sethu Sheeladevi[1,3*¶], John G Lawrenson[1¶],

Ramesh Kekunnaya[2&], Rahul Ali[3&], Rishi R Borah[3&], Catherine Suttle, [1¶]

1 – Centre for Applied Vision Research, Division of Optometry and Visual Science, City, University of London

2 – Child Sight Institute & Jasti V Ramanamma Children's Eye Care Centre, L V Prasad Eye Institute, Hyderabad, India

3 – Orbis International, India

***Corresponding author**

Email: Sheeladevi.sethu@orbis.org (SS)

Abstract

Early presentation for childhood cataract surgery is an important first step in preventing related visual impairment and blindness. In the absence of neonatal eye screening programmes in developing countries, the early identification of childhood cataract remains a major challenge. The primary aim of this study was to identify potential barriers to accessing childhood cataract services from the perspective of parents and carers, as a critical step towards increasing the timely uptake of cataract surgery.

Methods:

In-depth interviews were conducted using a pre-designed topic guide developed for this study to seek the views of parents and carers in eight Indian states regarding their perceived barriers and enablers to accessing childhood cataract services. A total of 35 in-depth interviews were conducted including 30 at the hospital premises and 5 in the participants' homes.

All interviews were conducted in local language and audio taped for further transcription and analysis. Data was organised with NVivo 11 and a thematic analysis was conducted utilising the Theoretical Domains Framework (TDF), an integrative framework of behavioural theories.

Results:

A total of 632 quotes were coded and a total of 68 themes were identified, including 23 related to barriers, 17 related to enablers and 28 themes represented both barriers and enablers covering 11 out of 12 TDF domains. TDF domains associated with barriers included: 'Environmental context and resources', 'Beliefs about consequences' and 'Social influences'. Reported enablers were identified in three theoretical domains: 'Social influences', 'Beliefs about consequences' and 'Motivations and goals'. This comprehensive TDF approach

enabled us to understand the parents perceived barriers and enablers to access childhood cataract services.

Conclusion:

This study has identified common barriers and enablers to accessing childhood cataract services that could be targeted in future interventions to improve timely uptake.’

Introduction

In cases of childhood cataract, early presentation for surgery is an essential first step in preventing associated visual impairment and blindness. The recommended age for congenital cataract surgery in children is congenital cataract is within the first six to eight weeks after birth for unilateral cases (1, 2), However, in India, cataract surgery in children is often delayed and a recent prospective study across India shows that the mean age at surgery for congenital and developmental cataract was 4 years and 8 years respectively (3). Delayed cataract surgery in children has a profound effect on visual outcomes (4, 5). In order to improve access, there is a need to understand the barriers and enablers associated with the access to childhood cataract services in India.

Access to eye care services for children depends on health care seeking behaviour (HCSB) of their parents and carers as well as the availability of eye care facilities in their community (6). In this context, HCSB means the recognition of symptoms, timely presentation to health facilities and compliance with effective treatment. All these factors will influence postoperative visual prognosis.

HCSB is influenced by numerous factors including educational level, maternal occupation, marital status, economic status, age and sex, health care costs, women's status, type and severity of illness, distance and physical access, and perceived quality of service provision (7). This wide range of factors indicates that the provision of education and knowledge at the individual level is not sufficient in itself to promote a change in behaviour

(8). Understanding health care seeking behaviours more comprehensively at the level of the individual, the family and the larger community is likely to have benefits in addressing the gaps in service utilisation.

A number of models exist to explain health care seeking behaviour (9) but these are often not easily applicable by health professionals who do not have a psychology background (10). The Theoretical Domains Framework (TDF) was designed to systematically identify and assess barriers to health care and has been used to assess behavioural patterns among both health professionals and patients (11-13). Behaviour change interventions are challenging and to achieve positive outcomes it is recommended that implementation strategies have a theoretical basis. Moreover, the interventions are more likely to be effective if they address the determinants (barriers and enablers) of the target behaviour (14).

It is important for programme planners to select a theory that is appropriate for the behavioural problem that they are trying to change with due consideration given to the setting/ population. The Behaviour Change Wheel (BCW), is a framework that provides programme planners with a 'comprehensive, coherent, and universal toolkit for intervention design' and can be used to guide the choice of an appropriate intervention to achieve given outcomes (15).

This study uses the TDF approach and components of the behaviour change wheel to identify barriers and enablers associated with the access to childhood cataract services in India. Based on these findings, various behavioural intervention functions are recommended.

Methods

The consolidated criteria for reporting qualitative research (COREQ) guidelines were followed (16). This study received approval from the School of Health Sciences Research Ethics Committee, City, University of London, and the institutional review boards of nine hospitals across India. Written informed consent was obtained from each participant individually.

Study Design

In-depth interviews were conducted using a pre-designed topic guide developed for this study (Appendix:1) The topic guide was developed with the intention of helping the researcher to explore the barriers and enablers in depth and to stay focused on the topic and ensure all important question were raised during the interview. However, the structure allowed the researcher sufficient flexibility to permit topics to be covered in an order most suited to the participants, to allow responses to be fully probed and explored and allow the researcher to be responsive to issues raised spontaneously by the participants (17).

Participants and setting

To capture a diverse cohort of patients with varied experiences, a stratified purposive sampling technique was used to select participants who had experience in accessing cataract services for their child in nine hospital locations across India. The details of the hospitals and locations has been reported elsewhere (1). The stratification was conducted according to the child's surgery status including children who:

- had their cataract surgery without any delay, (defined as surgery completed within three months from recognition of the condition)
- had been advised to undergo surgery but had not done so within a 3-month period.

Participants fulfilling the above two criteria were selected both from free and paying section at the participating hospitals. At each hospital location, 3 to 4 interviews were planned. Saturation in interview studies is generally reached before 20 interviews. Hence, the target sample size for this study was 30 interviews with people in the target age group. (18). Additionally, home interviews were conducted for children who had been advised to undergo cataract surgery but had not done so. For these home interviews, two hospitals (Sankardeva Nethralaya in Assam and Sadguru Nethralaya Chitrakoot in Madhya Pradesh) were selected based on logistic convenience. This cohort was identified from hospital records and for practical reasons the families residing within 15 kms of the hospital were selected from the list. Telephone enquiries were made to check their availability and their consent to participate in the interview. Based on the verbal consent the home visits were made, and written consent was obtained from each participant (parent or carer) before the actual interview.

Procedure

Data were collected from the participants between Nov 2015 and April 2016. All interviews were conducted in local languages (six different languages) for the convenience of the participants to express their feelings openly during the discussion. Interviews were conducted in a separate room at the hospital to ensure privacy and to avoid any

disturbances. Home interviews were conducted with pre-arranged appointments with the family according to their convenience.

The interviews were audio taped with prior consent for transcription. All the hospital interviews and the home interviews were conducted by the principal researcher (SS) and a support staff member from the hospital. In two locations language interpreters with local language (Assamese and Bengali) and community experience were selected by the principal researcher from local partner hospitals to assist with the interview. Each interview started with an introduction by the researcher and the parents were reassured that the interview was confidential and would not affect the treatment for their child currently or in future.

Data Handling and Analysis

Data Coding and Analysis

A verbatim transcript of each interview was prepared from the tape recording of the sessions and imported into NVivo 11 for data management and analysis (19). Transcription of all audio tapes was conducted externally by an independent company, and the researcher reviewed the transcripts for accuracy and completeness. The researcher was present in all the in-depth interviews and hence, it was possible to cross check with the field notes of each interview to ensure no omissions. However, no major changes were made to transcripts based on the researcher's perception, retaining the participants' meaning. In cases where the language interpreters were used, they were involved in verifying the transcripts. To maintain anonymity, names of the participants were removed from transcripts. The researcher (SS) read all the 35 interview transcripts multiple times before coding the transcripts independently using the Theoretical Domain Framework (TDF).

Development of Coding framework

Quotes representing factors that helped or encouraged the parents/ carers to access the services early were coded as ‘enablers’ and the quotes that were associated with factors contributing to surgical delay were coded as ‘barriers’. Every extracted statement was coded based on the 12 domains of TDF and its related component construct (20), either into one domain or into multiple domains. For example, one patient’s father stated: “*We felt very sad. She is so young and she has got cataract! What would happen if we get her married? Problems can arise. So, without delay surgery should be done”*. This was coded to both “Beliefs about consequences” (“what would happen…?) and “Social Influences” (related to marriage “Problems can arise…”) domains.

Both deductive and interpretive approaches were used to ensure all themes were coded. As part of interpretive analysis, the focus was given to sifting and sorting the data to thematically synthesise and to identify key domains and key emerging issues under each identified domain. Two factors were used to identify the “importance criteria” (21), key domains which are likely to be the most important for influencing the access to childhood cataract services.

1. Frequency (the number) of beliefs identified as barriers and enablers under each domain and elaboration (number of themes and sub themes) within each domain. Based on the overall percentage of the utterances (≥ 60% represents the top three domains, which is discussed in detail in the results section and ≥ 80% represents the top five domains), the domains are prioritised and summary of TDF domains are presented as Table1.

Table: 1 Summary of TDF domains for Barriers and Enablers

Barriers to access childhood cataract services*

TDF Domains	Rank order	Frequency of utterances	% of utterances
Environmental context and resources	1	77	24%
Beliefs about consequences	2	74	23%
Social influences	3	48	15%
Emotions	4	35	11%
Motivation and goals	5	27	9%
Knowledge	6	24	8%
Beliefs about capabilities	7	11	3%
Nature of behaviour	8	10	3%
Behaviour regulation	9	3	1%
Skills	10	4	1%
Social professional role and identity	11	2	1%
Memory, attention and decision processes	N/A	0	0%
Total		317	

Enablers to access childhood cataract services*

TDF Domains	Rank order	Frequency of utterances	% of utterances
Social influences	1	68	21%
Beliefs about consequences	2	67	20%
Motivation and goals	3	59	18%
Knowledge	4	40	12%
Beliefs about capabilities	5	22	7%
Emotions	6	21	6%
Environmental context and resources	7	17	5%
Behaviour regulation	8	16	5%
Social professional role and identity	9	9	3%
Nature of behaviour	10	6	2%
Skills	11	3	1%
Memory, attention and decision processes	N/A	0	0%
Total		328	

*The TDF domain accounting for top 70% of all utterances in either barrier or facilitator categories are presented above the double lines

2. The expressed importance within each domain. This was primarily a qualitative judgement made by the researcher based on her perception during each in-depth interview conducted in this research.

A sample of 20% of all interview transcripts were randomly picked and coded independently by another researcher (CS). The agreement between the two researchers was assessed in a meeting by reviewing all the codes assigned to each of the 12 TDF domains. The inter coder agreement was over 90% on all domains. All disagreements or uncertainties were discussed, and a final theme was agreed.

Results

A total of 35 in-depth interviews were conducted including 30 at the hospital premises and 5 in the participants' homes. Time duration for interviews ranged from 40 to 90 minutes. The interviews were held with both parents (n = 14), mother (10), father (6), grandparents (2) and family groups (3) in which a few other relatives along with the parents also participated in the discussion.

Based on the interview transcripts, a total of 632 quotes were coded and a total of 68 themes were identified, including 23 related to barriers, 17 related to enablers and 28 themes represented both barriers and enablers covering 11 out of 12 TDF domains. No quotes were consistent with the domain of 'memory, attention and decision processes'. Figure 1 shows the top five TDF domains and the themes identified under barriers and

enablers. A summary of the number of quotes coded under each TDF domain under barriers and enablers and an example quote is attached as annexure 2 and 3 respectively.

Figure 1: Top 5 TDF domains identified as Barriers and Enablers to access childhood cataract services.

Barriers to access of childhood cataract services

The three most important domains representing perceived barriers to childhood cataract services were found to be *environmental context and resources*, *beliefs about consequences* and *social influences*. Each of these are explained below, with examples.

1. **Environmental context and resources**

Most of the barriers identified by participants fitted this domain, and the most frequently cited barriers could be categorised under the following themes:

Economic reasons: The cost involved in seeking services was found to be the major barrier. Although the surgical services are available at no cost at the participating hospitals, the related costs such as travel, and lost wages may be significant. One participant stated: *I do labour job… It is labour job. If you go, you get (wages), if you don't go, you don't get...and I have two more children in the family… (Father, ref ID 16)*

Healthcare facility: Inadequate surgical eye care facility, lack of eye screening programs for children and the protocols and procedures followed at the hospitals were also identified as barriers within this domain. For example, the following comment reflects a lack of nearby surgical facility: *For eye problems people go to the doctors in nearby town. They would give*

medicines and a pair of spectacles… with spectacles one has to be happy… Other than that, there is no surgery service available close by. (Father, ref ID 4)

Environmental stressors: These include issues related to distance to the hospital, transportation and the time taken for appointments contributed as barriers for seeking care. Most of the children's eye care facilities were located in larger towns and cities making it necessary for the families outside of these locations to travel long distances to access the services. Given the nature of surgical service and the number of follow ups required for the child pre and post op, parents and carers reported many challenges with various environmental stressors. An example comment from one child's parents indicates difficulty reaching the healthcare facility for treatment: *They told [us] to come after 2 months... we have to see ... there is no time, expenses too and we have to close the shop we run... it gets more difficult for other two daughters to go to school (Parents, ref ID 12)*

2. **Beliefs about consequences**

Participants expressed concern about perceived consequences as barriers that contributed to delay in accessing cataract services. Most important themes under this barrier category included, consequences, beliefs, and attitudes. Within the theme of *beliefs*, traditional / cultural beliefs based on spirituality and old practices were also found to be a major impediment for accessing cataract services for the children. For example: *It happened since he was born. Now he is 4 years old. It is there since four years. We kept on thinking that it will go away, it will be cured. We kept on going to "babaji's place" [local priest]. People recommend this place to visit… to get cured. That's it … and the time kept passing on… (Father, Ref Id 13).*

Though some parents understand the consequences of delayed treatment, they may still delay accessing treatment due to a perception of other consequences such as negative outcomes post-surgery. One comment, for example, shows concern about harm related to the surgery: *If we tell him to bring something, he is unable to pick it up … I felt very sad but he is too small [young], so we didn't go for the surgery. If he rubs his eyes after surgery…he might get hurt himself. (Grandparents, ref Id 26)*

Lack of appreciation of the need for preventive care such as regular eye screening for early detection contributed to a delay in accessing cataract services in children, for example: *No…. we never thought the child should go for an eye check-up when there is no problem... Only if there is a problem, children should be taken for check-up. Otherwise not necessary. (Parents, Ref ID 10)*

3. **Social influences**

Social Influences also contributed significantly to the delay in accessing the services. The major themes identified as barriers under this domain were 'Social norms/ culture', 'Social Pressure' and Social support at the family level.

Cultural and societal norms dictate to some extent the relatively low priority given to vision and eye care for children. Social and peer pressure for the parents to try alternative forms of treatment resulted in delay in accessing cataract surgical services in children. For example: *Yeah, people said about Ayurveda treatment [Herbal treatment]. We had tried with Ayurveda in parallel and came to know that by applying Ayurveda medicines in the eyes*

cataract could be cleared. When we came for the surgery we received calls suggesting us to not to go for the surgery… (Parents, ref ID 18).

Pressure and a lack of empathy from peers acted as an emotional barrier among the children with cataract and their families. Parents in law played a major role in overall decision making for health care visits in several rural families. Unless they are convinced about the treatment they will not allow their grandchildren go for surgery even the mother is willing to do. For example, under the domain 'Social norms/ culture' a mother in rural area expressed it as: *I have accumulated the money for my son's operation … But, my in laws were not allowing us to go for surgery as everyone feels the child is too young... (Mother, ref ID 26)*

Enablers to access of childhood cataract services

The three domains into which most of the enablers cited by participants fitted were 'Social influences', 'beliefs about consequences' and 'motivation, goals and intention'.

1. **Social Influences**

The social structure of the family and the extended community had a significant impact on the decision-making processes around accessing cataract services. These included an individual's support in identifying or recognising the problem, help in arriving at an appropriate decision and most importantly the support extended either in accompanying the parent/carer to the hospital or looking after the young siblings when the parents are away receiving hospital care. Those interpersonal processes that can cause individuals to change their views, feelings, or behaviours are grouped under this domain.

The major themes identified as enablers within this domain are as follows: "Social Influences" are 'Social support', 'Social norms/ culture' and 'change agents within the community'. For example: a parent in rural area expressed it as:

In the rural areas people don't have money. So, major decisions are taken after the family members sit together and decide on where the money will be arranged from and how. Sometimes neighbours and / or relatives are also involved. (Parents, ref Id 3)

Many participants reported that deviations from social norms do occur. Some mothers had made the decision to take their child to the hospital when required even in rural communities where decision making of this kind is seen as a role for male members of the family. For example: *I take the decision as a mother and my husband doesn't say anything. Even in the neighbourhood as well men do not have time … men are busy with their work. Children's issues need to be looked after by their mothers. (Mother, ref Id 23)*

There were a few champions identified at the community level who are currently guiding the parents/ carers to seek appropriate care. They included the ASHA workers (Accredited Social Health Activist) appointed by the government to improve the health of rural communities. Significantly, parents whose children have already received care at the hospital were identified as champions of change within their communities.

2. **Beliefs about consequences**

Parents who realised the importance of eye treatment for their child were more likely to take their child for treatment. The major themes identified as enablers under this domain are 'Attitude', 'Reinforcement' and 'Value'. For example: …*We felt… but, then we thought it*

is about the eyes and eyes are everything. So, surgery is necessary. My elder brother also came with me. So, we quickly took the decision to go ahead with the surgery and admitted her. (Parents, Id 10)

The doctor – patient communication at the hospital played a pivotal role in enabling timely treatment, since they were in a position to reinforce the necessity of early treatment directly to the parents. For example: *The doctors told us that the surgery should be done immediately as with time the situation of the child would worsen. They showed me children aged as little as 4 months on whom surgery had been done and tried to convince me. They said that it is quite normal. After knowing all this, I became confident and went ahead with the surgery. (Father, Id 4)*

When the parent sees a benefit of treatment in their child, they are likely to give higher priority to spending time and money to make treatment possible. For example: *Doctor said that spectacle will be required for my child. So, I am getting it done here. There is no difficulty... Now my child is able to see… I will feel good to come back to the hospital…hoping that he would see better. (Father, ref Id 13)*

3. Motivation, goals and intention

The themes covered under this domain include the motivation of the parents and their intentions towards seeking services for their children. When the parents had clear intention to provide early treatment for their child, they accessed the services early despite having economic challenges in the families. The major themes identified as enablers under this

domain are ‘certainty of the intention’, ‘Intention’ and ‘Intrinsic motivation / service intention’. For example: *Everyone has different thoughts. I think that even if I am doing labour job, my child should not do this. He should do some better job. So I would forego my wages to bring my child for check up to make sure his eye sight is good. (Father, ref Id 5)*

When the parents had positive intention towards the importance of eye sight for their child, they accessed the surgical services early. For example: *It is a matter of the eyes. Every person will think that his child should be alright. (Father, ref Id 28)*

Families whose children have gained vision after cataract surgery had clear intention to spread the benefits of early surgery in children among their communities. For example: *When we go back with our daughter, many people come to see. So, we will tell them that if your child has any eye problem, you should go to the nearest eye doctor. (Father, ref Id 16)*

There are other themes outside of the top 5 domains, but which may be important enabling factors for accessing childhood cataract services. The referral slip provided during the community screening program ensures confidence and motivation to access hospitals in different towns as parents were informed about the hospital location and where exactly they need to report within the hospital. For example: *… if had come alone, I would have been confused… what to do, where to go …when I came here, I had a referral slip with me from the community screening, where it was written ‘contact counter no 4’…I went to the counter no 4, where the registration was done and then whatever was required at the hospital staff went ahead with their work and prepared the file. (Father, ref Id 5)*

Discussion

Summary of key findings

This in-depth qualitative study is the first to explore the perceptions of parents and carers towards accessing childhood cataract services from multiple regions in India. This is also the first study to use TDF (which includes constructs from 33 behaviour change theories) in the field of community eye care to identify barriers and enablers for accessing childhood cataract services. The current study identified four TDF framework domains as the most influential factors as reported by parents and carers: 1) 'Environmental context and resources'; 2) 'Social influences'; 3) 'Motivations and goals' ; 4) 'Beliefs about consequences'. Interventions that target these domains may be more likely to increase cataract surgery uptake in children.

A major concern reported by the parents and carers during the in-depth interviews related to the economic barriers to utilising the hospital services. Even when there is a possibility of free surgery at the hospital, the indirect costs associated with the treatment were reported to be a major barrier. Opportunity costs were a major issue as most of the parents had other family members including children and elders who depended on their income.

Previous research suggests that despite financial difficulties, health care utilisation is more likely if the illness is perceived to be either severe or life threatening (22). In the case of cataract, parents and carers in the community were generally aware of the development and management of cataracts in adults. In particular, most adult cataracts tend to be treated in late adulthood and once the cataract is mature (described as 'ripe' in local terminology). In the present study, the elders in the family had significant involvement in

decision making for health seeking behaviour. Those elders without the experience of childhood cataract may influence parents to delay surgery in children if they consider it benign and unlikely to have negative impact on vision.

However, economic issues did not deter parents from seeking surgery for their child if they were aware about the importance of early treatment and had access to a good health care facility. For example, parents with poor economic backgrounds expressed that, while they have no money available for cataract treatment, they were keen to raise money from other sources as they considered vision to be important for the better future of their child. This indicates the importance of creating awareness of the need for early treatment in this population. For example, the families need to be educated about the risk of losing vision permanently in children with cataract if the surgery is delayed.

Implications for policy and practice

One of the key findings from this study is that establishing community eye screening programs in rural areas can act as both a barrier and an enabler for accessing cataract services in children (Appendix 2). The qualitative interviews revealed that some parents were waiting for the community screening program to be organised in their vicinity to access free services for their children, despite being aware of the problem and the need for early surgery. The inability to pay for the surgical services mean that they wait for the screening camp to arrive in the villages. However, in case of surgical referrals from the community screening, the parents reported confident in accessing the referred facility as they were provided with all the details in the referral form given to them.

Although most carers accessed eye care centres after recognising the eye problem, a substantial minority chose to access traditional healers and other forms of care initially, potentially delaying the opportunities for optimal intervention. Previous research in developing countries about health seeking behaviour for childhood illness (23) has indicated that care seeking behaviour in resource poor settings is a hierarchical process, in which carers first seek inexpensive solutions before visiting a hospital. Cultural beliefs based on religion and superstition were found to be an important impediment in accessing surgical services.

Another important factor that influenced access to childhood cataract services in this study was the social support provided by family and relatives and the wider community as a source of information and guidance. Cataract surgery requires admission for a minimum of three days at the hospital and mostly these hospitals are located in major towns and cities. If the family has more than one child, the support system within the family becomes an important enabling factor for cataract surgery. Arrangements need to be made for a member (usually a grandparent) to look after the other siblings whilst the parents are away. Similarly, after surgery a support network is required to ensure good post-operative recovery and later follow-up in children.

Previous work suggests that any decisions regarding the child's health and access to care are made at the household level and that these decisions are largely influenced by household factors such as parents' educational and occupational exposures and mainly depend on the household income (24). A similar pattern was observed in the present research. Also,

parents-in-law played a major role in overall decision making in rural families and this is consistent with previous findings (25).

The present findings also indicate that the eye care professionals in rural areas and in smaller towns may give inappropriate advice to the parents about cataract surgery in children (Appendix 2). This would have caused delayed presentation for cataract surgery and is modifiable with continuous medical education programs to update the local practitioners' knowledge in rural areas as they play a crucial role in timely referral to tertiary centres for surgical services.

Although Knowledge was not identified as one of the top three domains in this research, it is important to note that most of the participants were not aware of the specific issues regarding childhood cataract. However, the prior experience of the family related to adult cataract may have had a greater influence in the parent's health-seeking behaviour. This finding contrasts with earlier studies, in which knowledge among the parents about children's eye diseases was generally low but they were aware of cataract in children (26, 27). This difference is due to the majority of the respondents in this study being from rural locations, where eye care service availability is very limited, whereas both previous studies were based on urban populations.

The findings of this research suggest that there are facilitators and barriers to childhood cataract services which are modifiable such as beliefs and consequences, social influences and knowledge, and this study is an important first step in establishing evidence as a basis for addressing issues on this topic. Further, this study has identified that there is a need to

modify the health seeking behaviour of the parents and carers to address the issues related to childhood cataract services.

There are four steps involved in developing a theory informed implementaion intervention for achieving positive health seeking behaviour among the target population (28). The first two of these involve identifying the specific behaviour and the target group and using the TDF approach to identify barriers and enablers that need to be addressed. Based on these two factors, the third step is to identify the intervention components that are feasible, relevant to the cultural context and defines the measures of behaviour change. This research has addressed the first two steps towards developing a theory information implementation. Based on the present finding this study has recommended various intervention functions using behavioural change wheel components of the COM-B model (15) to increase the timely uptake of cataract services in children Table 2. Programs implemented in wider communities should be developed that target specific behaviours that could bring changes in how childhood cataract and its treatment is perceived to increase the uptake of services by the communities. The impact of implementing such positive behavioural changes program in the community should be investigated using a robust experimental design.

Table 2 Recommended intervention functions for increasing the timely uptake of childhood cataract services in India

Barrier domains*	Details of Barriers	Target audience	COM-B components **	Recommended intervention functions *
Beliefs about Consequences	A belief that it is acceptable to delay cataract surgery in children<br>A belief that a visit to babaji (local priest) will cure the cataract in children	Parents and carers	Reflective motivation	Education and Modelling
Motivation and goals	No intention to take the child for any routine eye examination, citing time constraints	Parents and carers	Reflective motivation	Education, Incentivisation, and Modelling
Environmental Context and Resources	Economic constraints and limited the feasibility of travelling long distances to seek the treatment	Parents and carers	Physical opportunity	Environmental restructuring, Training, and Enablement
Social Influences	Parents were influenced by what their friends and families did and recommended.	Parents and carers	Social opportunity	Environmental restructuring, training and Enablement
Knowledge	A lack of knowledge of cataract in children, and lack of awareness about the preventive aspects and when to go for surgery.	Parents and carers	Psychological capability	Education

** - COM-B component stands for Capability (Physical capability or Psychological capability), Opportunity (Physical opportunity or Social opportunity), and Motivation (Automatic motivation or Reflective motivation)– Behaviour, represents source of the behaviours and is the core of the BCW

* Recommended intervention functions were identified by the Behaviour Change Wheel (BCW)

Strengths and weakness of the study

This is the first study to look systematically at the barriers and enablers for childhood cataract services in India using the TDF to identify theoretical perspectives associated with the identified issues. The respondents included in this research were selected from nine different geographical regions in India with broad cross-cultural representation. Also, the participants included parents, carers and other family members chosen from rural, urban and remote hilly and tribal areas to cover a range of perspectives among families in these regions.

The in-depth interviews were conducted with the parents and carers who had already accessed the hospital for childhood cataract surgery. The reported barriers and enablers from this study are therefore relevant to the members of the community who have accessed the services. Ideally, the barriers assessment should be focused at the community level from those who have not accessed the facility for their children's cataract as the associated barriers may be different in this group.

Most of the interviews were conducted at the hospital premises, either at the time of admission for surgery or during the follow up care. The hospital environment may have had an influence, for example parents and carers may have felt obliged to give favourable answers thinking that their response would have an effect on their child's care.
Another important limitation in this study is the involvement of language interpreters to facilitate the discussion in few locations. This has an inherent disadvantage on the flow and continuity of the discussion and may potentially impact on the interaction with the participants.

A deductive approach was used to code the data. All of the coded data were further analysed using an interpretive approach. An inductive approach would be appropriate in this context to identify new themes in the data, however no new themes emerged from these data, rather most of the data collected fitted well within the original TDF themes. However, the TDF domains did not fit the data perfectly in a few instances. For example, gender and religious beliefs were categorised under the domain Social Influence in this present study, but given the importance of these two aspects the framework did not allow full exploration of the related issues. This categorisation may have limited the extent to which these issues were explored in the interviews and in the analysis.

Future research

Childhood cataract surgery requires long term follow up and achieving a good visual outcome after cataract surgery is likely to depend at least in part on the post-operative follow up care in children. Achieving maximum uptake of follow up care is a continuous challenge even in adults for any chronic conditions and it is considered more challenging in children. The present research was focused on access to services and additional research is needed to assess the barriers and enablers associated with the access to follow-up care post childhood cataract surgery in different regions.

Conclusion

Our findings highlight that the TDF is considered as a useful approach providing a systematic, comprehensive, and theory-derived process to identify barriers to access childhood cataract services that can help identify target behaviours for change and inform

implementation strategies. Also, this study found that the TDF was a flexible approach that could be used across different settings and in different ways to understand planning and implementation of relevant activities.

Acknowledgement

The authors acknowledge the support of staff and management of all participant hospitals, parents and children who have contributed to this study.

1. Birch EE, Cheng C, Stager DR, Jr., Weakley DR, Jr., Stager DR, Sr. The critical period for surgical treatment of dense congenital bilateral cataracts. J aapos. 2009;13(1):67-71.

2. Birch EE, Stager D, Leffler J, Weakley D. Early treatment of congenital unilateral cataract minimizes unequal competition. Invest Ophthalmol Vis Sci. 1998;39(9):1560-6.

3. Sheeladevi S, Lawrenson JG, Fielder A, Kekunnaya R, Ali R, Borah RR, et al. Delay in presentation to hospital for childhood cataract surgery in India. Eye (Lond). 2018;32(12):1811-8.

4. Chak M, Wade A, Rahi JS, British Congenital Cataract Interest G. Long-term visual acuity and its predictors after surgery for congenital cataract: findings of the British congenital cataract study. Invest Ophthalmol Vis Sci. 2006;47(10):4262-9.

5. Taylor D, Wright KW, Amaya L, Cassidy L, Nischal K, Russell-Eggitt I, et al. Should we aggressively treat unilateral congenital cataracts? Br J Ophthalmol. 2001;85(9):1120-6.

6. Lei L. The impact of community context on children's health and nutritional status in China. Social science & medicine (1982). 2017;179:172-81.

7. Tipping G, Segall M. Health care seeking behaviour in developing countries: an annotated bibliography and literature review. 1995(Jul. [3],): 89 p.

8. Rimal RN. Closing the Knowledge-Behavior Gap in Health Promotion: The Mediating Role of Self-Efficacy. Health Communication. 2000;12(3):219-37.

9. Michie S, West R, Campbell BJ, H G. ABC of Behaviour Change Theories (Behaviour change) - An essential resource for researchers and practitioners. 2014.

10. Michie S, Johnston M, Abraham C, Lawton R, Parker D, Walker A. Making psychological theory useful for implementing evidence based practice: a consensus approach. Quality Safety in Health Care. 2005;14.

11. Burgess C, Wright AJ, Forster AS, Dodhia H, Miller J, Fuller F, et al. Influences on individuals' decisions to take up the offer of a health check: a qualitative study. Health expectations : an international journal of public participation in health care and health policy. 2015;18(6):2437-48.

12. Gray-Burrows KA, Day PF, Marshman Z, Aliakbari E, Prady SL, McEachan RR. Using intervention mapping to develop a home-based parental-supervised toothbrushing intervention for young children. Implementation science : IS. 2016;11:61.

13. Dyson J, Lawton R, Jackson C, Cheater F. Does the use of a theoretical approach tell us more about hand hygiene behaviour? The barriers and levers to hand hygiene. Journal of Infection Prevention. 2010;12(1):17-24.

14. French SD, Green SE, O'Connor DA, McKenzie JE, Francis JJ, Michie S, et al. Developing theory-informed behaviour change interventions to implement evidence into practice: a systematic approach using the Theoretical Domains Framework. Implementation science : IS. 2012;7:38.

15. Michie S, van Stralen MM, West R. The behaviour change wheel: a new method for characterising and designing behaviour change interventions. Implementation science : IS. 2011;6:42.

16. Tong A, Sainsbury P, Craig J. Consolidated criteria for reporting qualitative research (COREQ): a 32-item checklist for interviews and focus groups. International journal for quality in health care : journal of the International Society for Quality in Health Care. 2007;19(6):349-57.

17. Ritchie J, Lewis J, Nicholls CM, Ormston R. Qualitative research practice: A guide for social science students and researchers: Sage; 2013.

18. Francis JJ, Johnston M, Robertson C, Glidewell L, Entwistle V, Eccles MP, et al. What is an adequate sample size? Operationalising data saturation for theory-based interview studies. Psychol Health. 2010;25(10):1229-45.

19. QSR-International. NVivo Qualitative Data Analysis Software. 2013;Version 11.

20. Michie S, Johnston M, Abraham C, Lawton R, Parker D, Walker A. Making psychological theory useful for implementing evidence based practice: a consensus approach. Qual Saf Health Care. 2005;14(1):26-33.

21. Graham-Rowe E, Lorencatto F, Lawrenson JG, Burr J, Grimshaw JM, Ivers NM, et al. Barriers and enablers to diabetic retinopathy screening attendance: Protocol for a systematic review. Systematic reviews. 2016;5(1):134.

22. Russell S. Ability to pay for health care: concepts and evidence. Health policy and planning. 1996;11(3):219-37.

23. Nasrin D, Wu Y, Blackwelder WC, Farag TH, Saha D, Sow SO, et al. Health care seeking for Childhood Diarrhea in Developing Countries: Evidence from Seven Sites in Africa and Asia. The American Journal of Tropical Medicine and Hygiene. 2013;89(1 Suppl):3-12.

24. Pillai RK, Williams SV, Glick HA, Polsky D, Berlin JA, Lowe RA. Factors affecting decisions to seek treatment for sick children in Kerala, India. Social science & medicine (1982). 2003;57(5):783-90.

25. Pokhrel S, Sauerborn R. Household decision-making on child health care in developing countries: the case of Nepal. Health policy and planning. 2004;19(4):218-33.

26. Ebeigbe JA, Emedike CM. Parents’ awareness and perception of children's eye diseases in Nigeria. Journal of Optometry. 2017;10(2):104-10.

27. Senthilkumar D, Balasubramaniam SM, Kumaran SE, Ramani KK. Parents' awareness and perception of children's eye diseases in Chennai, India. Optometry and vision science : official publication of the American Academy of Optometry. 2013;90(12):1462-6.

28. French SD, Green SE, O’Connor DA, McKenzie JE, Francis JJ, Michie S, et al. Developing theory-informed behaviour change interventions to implement evidence into practice: a systematic approach using the Theoretical Domains Framework. Implementation Science. 2012;7(1):38.

Figure 1: Top 5 TDF domains identified as Barriers and Enablers to access childhood cataract services.

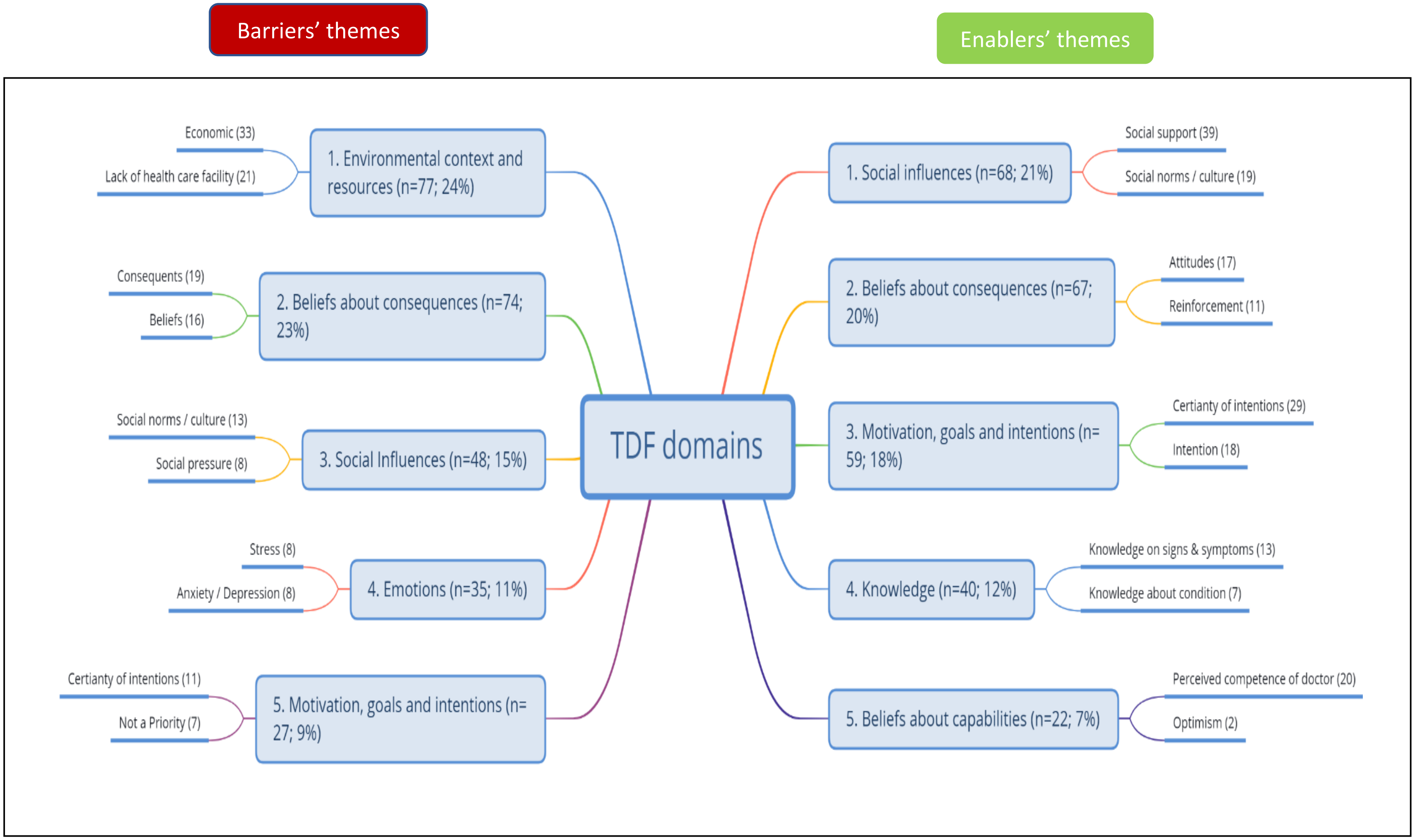

The number within brackets indicates the number of quotes identified under each domain and theme

Supplementary tables

Click here to access/download
Supporting Information
Appendix .docx
