# Supplementary material for: Barriers and enablers to access childhood cataract services across India. A qualitative study using the Theoretical Domains Framework (TDF) of behaviour change
Source: PLoS One. 2021 Dec 31;16(12):e0261308. doi: 10.1371/journal.pone.0261308 (PMC8719670; doi:10.1371/journal.pone.0261308)
Supplement: Attachment — Submitted filename: Response to Reviewers .docx [file pone.0261308.s007.docx]

| **Journal requirements** | **Response** |
| --- | --- |
| Please ensure that your manuscript meets PLOS ONE's style requirements, including those for file naming. The PLOS ONE style templates can be found at  <https://journals.plos.org/plosone/s/file?id=wjVg/PLOSOne_formatting_sample_main_body.pdf> and  <https://journals.plos.org/plosone/s/file?id=ba62/PLOSOne_formatting_sample_title_authors_affiliations.pdf>. | The manuscript file is updated as per the journal style requirements |
| Please ensure that all references are cited correctly. For example, we note that in the Methods, the sentence " The details of the hospitals and locations has been reported elsewhere (1)" seems to refer to the wrong citation. | All references are now cited correctly. |
| Thank you for stating the following financial disclosure: "NO"  At this time, please address the following queries:  a) Please clarify the sources of funding (financial or material support) for your study. List the grants or organizations that supported your study, including funding received from your institution.  b) State what role the funders took in the study. If the funders had no role in your study, please state: “The funders had no role in study design, data collection and analysis, decision to publish, or preparation of the manuscript.” c) If any authors received a salary from any of your funders, please state which authors and which funders. d) If you did not receive any funding for this study, please state: “The authors received no specific funding for this work.”  Please include your amended statements within your cover letter; we will change the online submission form on your behalf. | We have included the details in the cover letter |
| Thank you for stating the following in your Competing Interests section: "No authors have competing interests"  Please complete your Competing Interests on the online submission form to state any Competing Interests. If you have no competing interests, please state "The authors have declared that no competing interests exist.", as detailed online in our guide for authors at <http://journals.plos.org/plosone/s/submit-now>  This information should be included in your cover letter; we will change the online submission form on your behalf. | We have included the details in the cover letter |
| In your Data Availability statement, you have not specified where the minimal data set underlying the results described in your manuscript can be found. PLOS defines a study's minimal data set as the underlying data used to reach the conclusions drawn in the manuscript and any additional data required to replicate the reported study findings in their entirety. All PLOS journals require that the minimal data set be made fully available. For more information about our data policy, please see <http://journals.plos.org/plosone/s/data-availability>.  Upon re-submitting your revised manuscript, please upload your study’s minimal underlying data set as either Supporting Information files or to a stable, public repository and include the relevant URLs, DOIs, or accession numbers within your revised cover letter. For a list of acceptable repositories, please see <http://journals.plos.org/plosone/s/data-availability#loc-recommended-repositories>. Any potentially identifying patient information must be fully anonymized.  Important: If there are ethical or legal restrictions to sharing your data publicly, please explain these restrictions in detail. Please see our guidelines for more information on what we consider unacceptable restrictions to publicly sharing data: <http://journals.plos.org/plosone/s/data-availability#loc-unacceptable-data-access-restrictions>. Note that it is not acceptable for the authors to be the sole named individuals responsible for ensuring data access.  We will update your Data Availability statement to reflect the information you provide in your cover letter. | We have included all the coded narratives and the themes assigned using the TDF approach from the 35 interview transcripts as a supplementary file.  This manuscript is part of a PhD thesis where all of the relevant data may be found. The thesis is freely available at <https://openaccess.city.ac.uk/id/eprint/20553/> |
| **Changes highlighted in the pdf document** |  |
| in eight Indian states (Page no 8) | This wording has been changed to ‘…in nine geographic locations across eight states in India.’ Please see page 2. |
| Is it childhood cataract care services? (page no 10) | We feel that the appropriate wording for this is ‘childhood cataract services’, and we left the wordings as it is.  Please see page 4. |
| Is the sample selection appropriate for studying barriers to access, as you are only considering patients who overcame those barriers and accessed care (page no 12) | We agree it is ideal to include subjects who have not accessed the services and this is one of the limitations, which is addressed in the discussion. However, we have identified the barriers that were faced by those who accessed services, and enablers that may facilitate their access. Different barriers may be faced by those unable to access cataract services and identification of those barriers would require a separate study. This point is discussed as part of the discussion, please see page 27. |
| what does diverse cohort mean? please check and edit (page no 12) | We have rephrased the sentence as follows to explain the concept of a diverse cohort:  ‘A stratified purposive sampling technique was used to ensure that the participants included those at different socio-economic levels and with experience of accessing cataract services for their child in a range of hospitals across India.’ Please see page 7. |
| Please check the reference (page no 12) | Reference no 3 has been replaced, please see page 7. |
| Not clear, what does free means? (page no 13) | Sentence rephrased as’ ‘paying’ and ‘non-paying’ sections in the hospital. Please see page 7. |
| What is this target age group? You have no where mentioned the same ( please define the same in methods if applicable or repharse) (page no 13) | Sentence rephrased as ‘whose children received cataract surgery’ (page 7). |
| Please pay attention to punctuations and full stops (page no 13) | These corrections have been made. |
| Who were interviewed, children or parents, how many in hospital and how many at home? (page no 13) | Only parents / carers /grandparents were interviewed. Thirty interviews were conducted in hospitals and 5 at home locations. The interviews were held with: i). both parents (n = 14) ii). mother only (n = 10) iii). father only (n = 6) iv). grandparents (n = 2) and v). family groups (n = 3). For the latter, other relatives along with the parents also participated in the discussion. See page 12. |
| Whose logistic convinience? participant or the researcher? why only disclosing the names of two hospitals? please keep it uniform (page no 13) | The term ‘logistic convenience’ in this context means to facilitate the organisation of the interviews and does not refer specifically to the researcher or the participants.  As suggested, we have removed the names of the two hospitals and rephrased as: ‘For these home interviews, two geographic locations were selected using purposive sampling to obtain different perspectives from rural and semi urban areas.’ Ref page no 7-8. |
| How did you manage to remove the selection bias ? (page no 13) | For the home visits, case selection was based on children who have been recommended for cataract surgery in the last 6 months but could not complete the surgery and whose residence is within 15 kms radius from the hospital. This list was auto generated from the medical records of the hospital and neither the researchers nor the hospital staff had any influence on the list. However, the interviews were conducted with the families who confirmed their availability when the field team contacted them over their mobile numbers registered with the hospital.  We have included the following paragraph in the strengths and weaknesses section on how the possibility of selection bias is addressed while using the purposive sampling method. See page no 27.  ‘In this study purposeful sampling method was used and as defined by Palinkas et al. This method is widely used in qualitative research for the identification and selection of information-rich cases who have the relevant experience and are willing to participate and communicate experiences in a reflective manner. <https://www.ncbi.nlm.nih.gov/pmc/articles/PMC4012002/>  However, the challenge in using this sampling strategy is that the range of variation in a sample is often not known at the beginning of a study or may be incorrectly assumed [32]. However, in this study maximum variant samples were used to obtain varied experiences in utilising the services.  All parents or carers bringing their child to each of the included hospitals were eligible for inclusion. Selection bias was therefore not introduced at the point of recruitment. The hospitals were selected, and our results may have differed if a different set of hospitals had been included.’ |
| Is this a duplication? (page no 14) | Yes, and it is removed now. Page 9. |
| Reference 1 (page no 34) | References are verified and updated to meet the journal style |
| Reference 18 (page no 35) | References are verified and updated to meet the journal style |
| **Reviewer 1** | **Response** |
| *General* |  |
| There needs to be consistency in writing “healthcare” and “health care”, choose one. My personal preference if the former. | This term is now ‘healthcare’ consistently throughout the document. |
| *Introduction* |  |
| Line 3 of Introduction: “…is congenital cataract…” should be removed as it's a typo. | This typographical error is now corrected, see page 4. |
| Last paragraph of introduction has a typo in the first sentence second line of that paragraph. The word “…the…” should be removed (i.e ‘…the access to childhood cataract services…’ should read as ‘…access to childhood cataract services…’. | This error has been corrected, see page 6. |
| *Methods* |  |
| Considering that this is a qualitative study the researchers should present statements on reflexivity and positionality of the authors. | The following statement has been added to the manuscript to address this point (page 8-9): ‘The first author, who led the data collection and analysis, is female, was born and raised in India and had worked in the field of rural eye care for several years and thus was able to understand the local context and cultural issues around it. The study was conducted as part of the author’s PhD research, and her preparation for the qualitative data collection in this study was gained as part of PhD training and in her prior role in community eye health research. The author had no prior relationship with the study participants.  To minimise the possibility of the first author’s ethno-racial background and cultural reflexivity influencing her interpretations of the data, a second author of United Kingdom origin independently coded the transcripts.’ |
| There is a mention of stratified purposive sampling, it would be ideal to mention or depict the sampling frame in a figure (i.e. what were the stratas?). | Participants were grouped according to the type of service, paid or unpaid, at the hospital. Under each of the payment categories, the participants were grouped according to any delay in surgery (within or beyond the advised period).  We have included the following details on stratification, see page 7.  The stratification was conducted according to the child’s surgery status including children who:   - had their cataract surgery without any delay, (defined as surgery completed within three months from recognition of the condition) - had been advised to undergo surgery but had not done so within a 3-month period.   Participants fulfilling the above two criteria were selected both from paying and non-paying patient categories at the participating hospitals. |
| You mention the use of 6 local languages. Which are those languages? Were interpreters the ones running the interviews? | The following six languages were used: Malayalam, Telegu, Hindi, Marathi, Bengali and Assamese. All the hospital interviews and the home interviews were conducted by the principal researcher (SS) and a support staff member from the hospital. In most cases, the principal researcher was able to communicate with the participants in their first language. In only two locations, Assamese and Bengali language interpreters with local community experience assisted with the interview. Please see page 8. |
| **Reviewer 2** | **Response** |
| *Background* |  |
| They must provide a little more detail about the Theoretical Determinants Framework in the background and introduction section. Has this framework been used in other public health settings? Has it been used in health care seeking behaviour settings? | We have included more details on TDF in the introduction as shown below: See page 5.  ‘The theoretical domains framework (TDF) was developed to make theories of behaviour and behaviour change more accessible to a range of users. The TDF has been cited in numerous peer reviewed publications focusing on behaviour patterns of health professionals and on patient uptake of healthcare services [11-13]. This framework has been used to analyse barriers and enablers in uptake of retinal screening among patients with Type 2 diabetes, [14] diabetic retinopathy screening attendance [15] and to understand professionals’ perspectives on depression in a vision rehabilitation setting [16].’ |
| *Methods* |  |
| What is the methodology adopted? This is not clear. Have the authors used grounded theory approach, narrative inquiry, phenomenology or case study? Have they taken an ethnographic approach? This methodological declaration is important and will help understand the rigor of the qualitative methodology | The TDF is intended for use by researchers and practitioners from many disciplines. Whilst users do not necessarily need expertise in using particular theories, a good understanding of the domains and the theoretical constructs each represents are recommended to aid interpretation of data. Data can be analysed deductively, using the TDF to generate the framework for a content analysis and, inductively, generating themes that can then be considered in relation to domains. <https://implementationscience.biomedcentral.com/articles/10.1186/s13012-017-0605-9>  We have described the methodology as follows in the methods section (page 10):  ‘Both deductive and inductive approaches were used to ensure all themes were coded. As part of inductive analysis, the focus was on sifting and sorting the data to thematically synthesise and to identify key domains and key emerging issues under each identified domain.’ |
| It is important to have a reflexivity note in the methods section. In this note the researchers must position themselves in the research context and declare their biases, opinions, experiences and provide reflection on how these will impact on the research findings. | The following statement has been added as part of methods (page 8):  ‘The first author, who led the data collection and analysis, is female, was born and raised in India and had worked in the field of rural eye care for several years and thus was able to understand the local context and cultural issues around it. The study was conducted as part of the author’s PhD research, and her preparation for the qualitative data collection in this study was gained as part of PhD training and in her prior role in community eye health research. The author established no relationship with the participants.  To minimise the possibility of the first author’s ethno-racial background and cultural reflexivity influencing her interpretations of the data, a second author of United Kingdom origin also independently coded the transcripts.’ |
| Most interviews have been conducted at the facility setting. There is a need to reflect on what kind of influence this choice of setting of the interviews will have on the findings. | As the reviewer points out, it is possible that participants’ responses were influenced by the fact that they were being asked within the hospital setting. Participants may have been influenced, for example, by concerns that their comments may affect their child’s care. The interviews were conducted by the first author and a member of hospital support staff, so from the participant’s perspective hospital staff had direct access to their comments. As part of the information provided to participants, they were assured that their participation in this research would not affect their child’s treatment at the hospital. However, responses may have been influenced by the hospital location and (in hospital and home interviews) the presence of a hospital staff member. This potential limitation is discussed in the Discussion section. Please see page 27. |
| Were any attempts made to triangulate the data obtained? There doesn’t seem to be any attempt at respondent triangulation, method triangulation. There is analytical triangulation reported, but other attempts at triangulation must also be presented. | Methodological triangulation was achieved using two qualitative methods, interviews and field notes. Details are provided in the Methods section as shown below. Ref page no. 9  ‘Transcription of all audio tapes was conducted externally by an independent company, and the researcher reviewed the transcripts for accuracy and completeness. The researcher was present in all the in-depth interviews and hence, it was possible to cross check with the field notes of each interview to ensure transcription accuracy.’  In addition, two investigators were independently involved in coding, to increase validity of the coding to TDF domains (deductive coding ) and identification of themes (inductive coding) (see page 12). |
| It is not clear why the authors chose to use in depth interviews and not focus group discussions for data collection. This choice must be justified | We have added the following explanation in the Methods section to clarify this point (please see page 6).  ‘This study aimed to understand barriers which may in some cases be unique to the individual. Barriers relating to financial and social factors may be difficult for parents to discuss in a group, so in-depth individual interviews were conducted using a pre-designed topic guide developed for this study’ |
| How is the presence of a translator during the interviews likely to influence the interviews. This must be reflected on. | A translator was present in some of the interviews. As the reviewer points out this means that an additional member of hospital staff was present during those interviews. The presence of an interpreter may have made participants less willing to voice any concerns. In addition, it is possible that the translation itself may have altered the meaning or emphasis in the participant’s words, and thus may have affected the results. However, the person engaged for transcribing the interviews, who was unrelated to this research, was able to capture the exact information conveyed by the participants.  This is considered as one of the limitations and reflected in the discussion section. See page 28.  We have added the following explanation in the Methods section to clarify further (please see page 9).  ‘For each interview, the transcriber was also a translator if the interview was not in a language known to the principal researcher, providing an additional check of the interviewee’s meaning.’ |
| A hospital support staff was also present during the interviews. This must be reflected on. How will presence of a hospital staff during the interviews influence the findings. It is likely that some barriers related to hospital services may not be presented. | In the subject information sheet assurance was given to the parents that their participation in this research would not affect their child’s treatment at the hospital. However, the presence of a member of hospital staff may have tempered responses and we have addressed this point as a limitation of the study. Please see page 28. |
| *Analysis* |  |
| A strong positivist and quantitative approach has been used in what is a mainly interpretivist paradigm of research. The authors have attempted to count and quantify the frequency of various domains of the framework. They have concluded that this frequency across the interviews is likely to indicate its importance. This assumption has many problems. For example for this assumption to be true, the sampling must be representative and the sample size must meet the requirements of statistical power and confidence. But the sampling, sample size and other considerations are for qualitative analysis, which does not take quantification into consideration. Therefore applying estimates and generalizing them as indicating importance of a particular dimension is problematic. A priori declaration of the ontological and epistemological position of the paper will help resolve this issue. This paper works on a realist ontological position with a subjectivist epistemological approach. However, the analysis violates this position. I strong recommend removing quantification and reporting of estimates of importance of various dimensions. | The TDF approach is a validated method to systematically assess the barriers and enablers in accessing childhood cataract care services. This method of quantification is commonly used in studies using the TDF approach which may be found at the following links:  <https://bmchealthservres.biomedcentral.com/articles/10.1186/s12913-020-05215-4/tables/2>  <https://www.ncbi.nlm.nih.gov/pmc/articles/PMC3522997/>  <https://implementationscience.biomedcentral.com/articles/10.1186/s13012-016-0416-4#Tab5>  The frequency of themes is calculated to understand the number of participants across all the interviews, whose responses contributed to that theme and the elaboration which is number of themes per domain.  Based on this we have included the following description in the method section and revised the table 1 and included a column on elaboration: Ref page no 10 – 12.   1. Frequency (the number) of beliefs identified as barriers and enablers under each domain and elaboration (number of themes and sub themes) within each domain. 2. The expressed importance within each domain. This was primarily a qualitative judgement made by the researcher based on her perception during each in-depth interview conducted in this research e.g spontaneity of themes (whether the theme occurred spontaneously or was elicited by a question in the topic guide).   Moreover, we used systematic purposive sampling to obtain maximum variant responses. Sampling used in this study is representative and achieved data saturation. |
| The inter-rater agreement statistics is also problematic. Such quantifications are beyond the scope of such a data. | The published literature using the TDF approach recommends a reliability check between the coders. For example, Patey et al reported inter-rater agreement in a TDF study Patey et al. Implement Sci. 2012;<https://www.ncbi.nlm.nih.gov/pmc/articles/PMC3522997/>  The reviewer’s comment does not provide us with enough information to understand why they feel that inter-rater agreement is not appropriate. We would be happy to look at this again if further information were provided. |
| The authors have used a pre-existing framework and have looked for these themes in the data. This is largely an deductive approach. There is no justification for why they did not do a inductive method of theme building. | TDF is a validated approach that has been widely used to assess various barriers and enablers systematically. In the TDF approach, the themes are generated through an inductive approach after assigning to TDF domains using a deductive approach. Please see page 10. |
| *Results* |  |
| In the results section, the authors should provide richer descriptions of each theme with more elaborate verbatim quotes. | We have included two detailed tables as an appendix including all verbatim quotes and the themes assigned to each of them from the 35 transcripts are included as a supplementary file. Some of the quotes in the supplementary file are referred-to in the discussion section. Please see page number 22 and 23. |
| *Discussion* |  |
| In the discussion section the authors have reported that they did not have any new themes that emerged from the data other than the framework that they have used. This is not possible. There is always scope for new themes to emerge from qualitative data. | As the author points out, new themes could always emerge from such data. Our statement that the data did not show any new themes does not disagree with this fact. We recruited until thematic saturation was reached i.e. no new information across TDF domains was provided in subsequent interviews. To clarify data saturation (in which themes are repeated but no new themes are raised) we have added the following paragraph in the Methods section on data saturation:  ‘Data saturation was reached when no new information on barriers and facilitators influencing access to cataract services across TDF domains was provided in subsequent interviews.’ Please see page 7.  In addition, the following paragraph is added to the discussion section describing how all themes fitted well within the TDF framework.  Data were initially analysed deductively, using the TDF to generate the framework for an inductive analysis that generated themes within TDF domains. ‘Whilst the TDF provides a useful and comprehensive theoretical approach to identifying influences on behaviour, if applied too rigidly there is a risk that non TDF-related participant perspectives could be missed. We attempted to reduce this risk by applying a more flexible inductive analytical approach to ensure that potential themes that could not be coded to the TDF were not lost. We have added acknowledgement and discussion of this important point on page 27 -28. |
