# Supplementary material for: Barriers and enablers to access childhood cataract services across India. A qualitative study using the Theoretical Domains Framework (TDF) of behaviour change
Source: PLoS One. 2021 Dec 31;16(12):e0261308. doi: 10.1371/journal.pone.0261308 (PMC8719670; doi:10.1371/journal.pone.0261308)
Supplement: Attachment — Submitted filename: Response to reviewer final.docx [file pone.0261308.s008.docx]

Reviewer’s comment:

Thank you for revising the manuscript and responding to the comments in detail. I am not convinced with the response to the questions related to analysis. Firstly the research paradigm used in this study is a social constructivist paradigm of understanding experiences, barriers, facilitators. The epistemological approach is subjective, using qualitative interviews. However the analysis method presents counting and quantification, which is a technique used in positivist approaches with objective epistemology. This is a conflict between the paradigm of research and analytical approach. The response that you have given is evidence of how this kind of quantification of the TDF framework is used in previous studies. Please provide a theoretical description of how such a positivist analytical approach is compatible with the social constructivist paradigm of research. It is for the same reasons that I also object to an inter-rater reliability. Inter rater reliability is a statistical method that follows a positivist paradigm. As this issue puts the core issue of validity of the findings of the paper into question, I would require a more detailed and theoretical response to this question.

Response to the reviewer:

We thank reviewer 2 for raising these concerns. In our previous response, we provided examples of research in which qualitative data are quantified but the reviewer rightly points out that we did not justify our use of this approach and asks for a theoretical description.

We quantified the number of utterances aligned with each domain as an indicator of the expressed importance of that domain. This was expressed in the manuscript as a rank order of importance. This approach is similar to the Framework Method that is widely used to support thematic (qualitative content) analysis. The framework method provides a systematic model for managing and mapping interview data to facilitate thematic analysis (*Ritchie J, Lewis J: Qualitative research practice: a guide for social science students and researchers. 2003, London: Sage*). We agree with the referee that since sampling in qualitative research is purposive in order to understand experiences, views or behaviours across a data set, attempts to quantify qualitative data (e.g. “13 out of 20 participants said X) is inappropriate. However, we feel that our method of analysis is still consistent with the subjective epistemological perspective required for qualitative analysis and reflects current guidance for applying the TDF in qualitative research. We therefore feel that it does not compromise the validity of the research findings. Nonetheless we have removed all references to quantification of the data in the main body of the text and have moved Table 1 reporting the rank order of TDF domains, to a supplementary appendix.

Similarly, we appreciate that formal evaluation of inter-coder reliability (ICR) is a controversial topic in the qualitative research community <https://journals.sagepub.com/doi/10.1177/1609406919899220>. Our view is that reporting of ICR is useful for convincing diverse audiences of the trustworthiness of the analysis. However, we are happy to remove the ICR analysis from the manuscript.
